# Supplementary material for: The interactome of intact mitochondria by cross-linking mass spectrometry provides evidence for coexisting respiratory supercomplexes
Source: Mol Cell Proteomics. 2017 Dec 8;17(2):216–32. doi: 10.1074/mcp.RA117.000470 (PMC5795388; doi:10.1074/mcp.RA117.000470)
Supplement: Supplemental Data [file supp_RA117.000470_133922_0_supp_23978_fzffwf.zip › spectra_annotation/mito_DR_spectra_annotation/262-1-2-1-2-1.pdf]

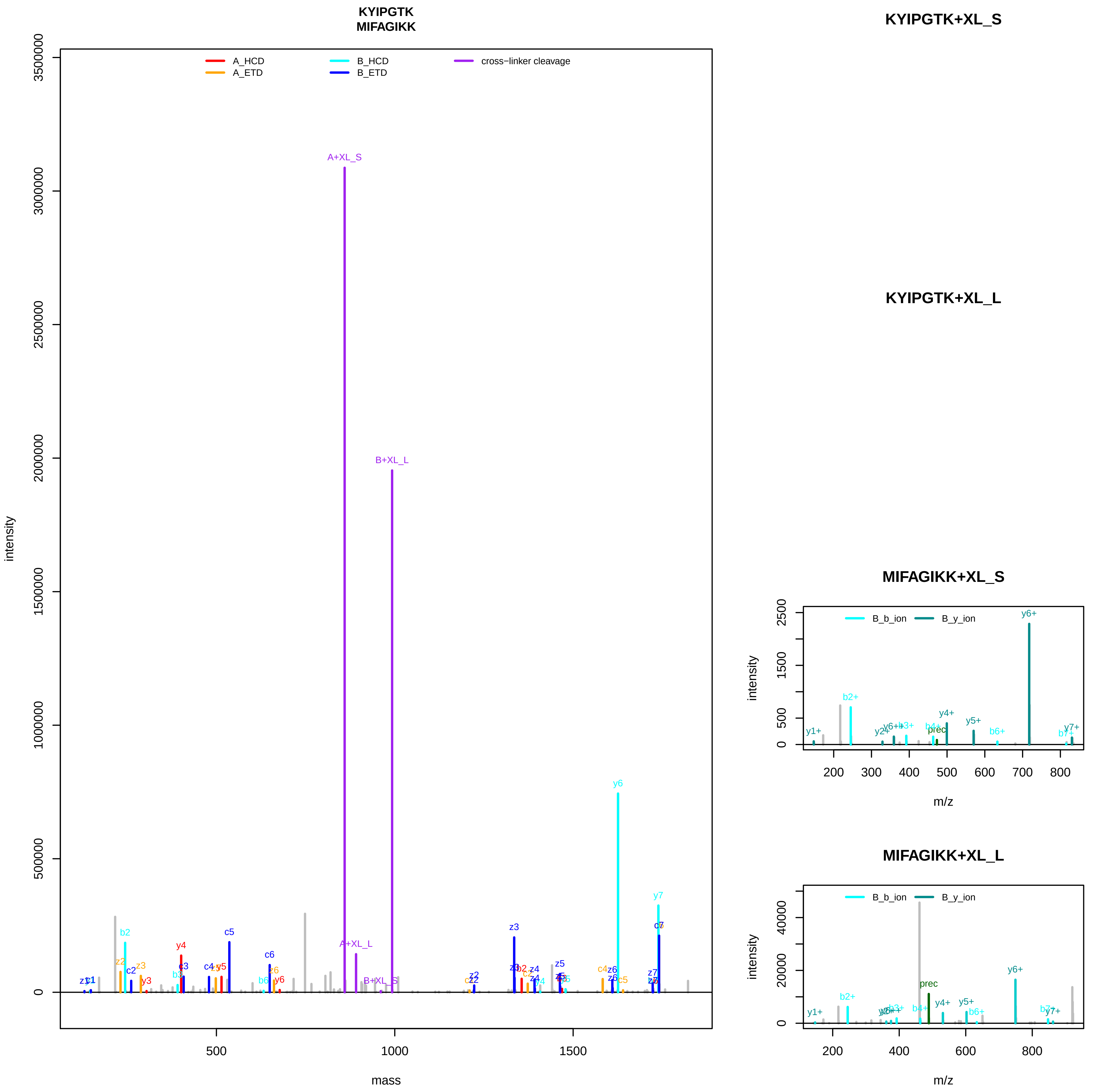

KYIPGTK
MIFAGIKK
A_HCD
A_ETD
B_HCD
B_ETD
cross−linker cleavage
A+XL_S
B+XL_L
A+XL_L
B+XL_S
y6
y7
c7
z3
c5
b2
y4
c6
c3
c4
y5
z6
c4
c5
z2
z3
z4
z5
z7
y3
y6
z1
c1
c2
z2
z3
b6
intensity
mass
0
500000
1000000
1500000
2000000
2500000
3000000
3500000
500
1000
1500
KYIPGTK+XL_S
KYIPGTK+XL_L
MIFAGIKK+XL_S
B_b_ion
B_y_ion
y6+
b2+
y4+
y5+
b6+
y7+
y1+
y2+
b3+
b4+
prec
intensity
m/z
0
500
1500
2500
200
300
400
500
600
700
800
MIFAGIKK+XL_L
B_b_ion
B_y_ion
y6+
b2+
prec
y4+
y5+
b6+
y7+
y1+
b4+
intensity
m/z
0
20000
40000
200
400
600
800
